# Easy Access, Difficult Consequences? Providing Psychiatric Patients With Access to Their Health Records Electronically

**DOI:** 10.3389/fpsyt.2019.00917

**Published:** 2019-12-16

**Authors:** Gillian Strudwick, Anthony Yeung, David Gratzer

**Affiliations:** ^1^Centre for Addiction and Mental Health, Toronto, ON, Canada; ^2^Institute of Health Policy, Management, and Evaluation, University of Toronto, Toronto, ON, Canada; ^3^Department of Psychiatry, University of Toronto, Toronto, ON, Canada

**Keywords:** psychiatry, OpenNotes, medical records, electronic health record, mental health

Should psychiatric patients have access to their clinical notes (often referred to as OpenNotes)? For decades, mental health providers have debated this. Some have argued that open access to notes will undermine the therapeutic rapport, and possibly undermine care itself (1). Others have suggested that involving patients in their care decisions must include access to their records; in 2014, an editorial called on providers to “show patients their mental health records” (2). Following this publication, several psychiatrists opened up their clinical notes to patients electronically through patient portals and have been providing access ever since (3). Despite initial concerns from providers about what opening up their notes might to do therapy and care, these concerns have generally not been realized (4, 5). We believe that more effort needs to be made to support providers in feeling more comfortable opening up their notes.

Although patient portals are increasingly found in other areas of medicine, they are not available to the same extent in psychiatry. Progress has been made both in terms of: 1) the number of organizations that have opened up their psychiatric notes (6); and, 2) our understanding of the impact of opening up notes to both patients and providers (7, 8). Yet, in our opinion, the uptake of “opening up” mental health clinical notes remains slow. In large part, we believe that this is due to the ongoing discomfort and concerns that providers have had (9).

A recent study has shown that working in psychiatry is a predictor of discomfort in providing clinical notes to patients, with psychiatrists and those working in acute settings being particularly concerned (9). This does not come as a surprise as we are aware of the sensitive topics discussed during clinical encounters, and the stigma often associated with mental illness. In our experience talking to providers about this topic, we have received mixed feedback from those that have not yet opened up their notes. There are providers who see the merit in opening up clinical notes to support patient empowerment, and providers who are opposed to the idea (often adamantly) due to a number of clinical concerns. These concerns include possible documentation changes that influence the clinical utility of the notes (note “sanitization”); patients reading their notes in unsupported environments and becoming upset; providers answering more questions after hours; patients not understanding the content of their notes; clinical interactions taking more time as questions related to note content are discussed; concerns about providers being subjected to violence; and, the list goes on (5, 8–10).

Since there have now been implementations of psychiatric patient portals with OpenNotes in numerous organizations, the question is now: what *have* the experiences been of providers? Generally speaking the literature has shown that the concerns of providers who are less comfortable with the idea of opening up their notes, have not been realized (4, 5). Although providers are concerned about a number of topics pre-implementation, the post-implementation reality seems incongruent with these initial concerns. One study showed that instead of the extensive time that providers worried about explaining the content of notes to patients, only about 15% of the time did patients even mention if they read their notes. In one study, patients who had access to their notes showed improvement in their self-reported mental health recovery scores, attended more appointments, and requested information less often from the health records department (possibly because they had access to the information they needed through the portal) (11). In this study, patients were provided a portion of their notes, and requested of the study researchers, increased access to the remaining notes in their record.

Although there is growing evidence that suggests that the concerns about opening up clinical notes to mental health patients may be unwarranted (and acknowledge that there may be some scenarios where granting access may not be as appropriate), we do believe that providers should be given more support with regards to how to make the transition to opening up their clinical notes. We believe it is important to validate concerns, yet identify opportunities to support providers in opening up their notes as comfortably as they can. We recommend that organizations use a toolkit developed on this topic by the OpenNotes team in Boston (12). In this toolkit, suggestions and vignettes for documenting sensitive topics are provided and could be utilized during training sessions. Organizations may wish to use a phased approach to open up notes. This could be done in a few ways. Certain note types (e.g., outpatient areas first) could be made available right away, and the remaining notes types made available at a later time. Another solution could be enrolling a small number of patients initially to ease providers into the process. As well, there have been initial successes documented in the literature when veterans have been provided web-based education related to reading their mental health notes online (7). Support for patients that does not rely on providers, could be considered.

Although privacy laws, consent norms and the specific legal considerations vary from country to country, patients in numerous countries have had the right to access their health record for a number of years now. However, in many organizations it's been challenging and sometimes expensive to do so, and therefore providers aren't accustomed to having patients read their notes. These same consent and legal implications that are present in most organizations today remain when electronic access is granted.

As healthcare moves toward patient empowerment and autonomy, the OpenNotes movement is here to stay. Although mental health records do have unique concerns that should be considered, there are tools and strategies to help providers navigate this. It is time to make open medical records a reality for all patients, and to support providers in this transition.

## Author Contributions

GS and DG conceptualized the commentary. The initial draft of the opinion was written by GS with substantive editing and inputs from DG and AY.

## Conflict of Interest

The authors declare that the research was conducted in the absence of any commercial or financial relationships that could be construed as a potential conflict of interest.

## References

[1] Reidbold S OpenNotes: Good intentions gone awry [Internet]. Psychology today. (2014). [cited 2019 Apr 28]. Available from: https://www.psychologytoday.com/us/blog/sacramento-street-psychiatry/201408/opennotes-good-intentions-gone-awry.

[2] Kahn M Bell S Walker J Delbanco T Let’s show patients their mental health records. J Am Med Assoc. (2014) 311(13):1291–2. 10.1001/jama.2014.1824 24691603

[3] OpenNotes OpenNotes History [Internet]. (2014). [cited 2019 Apr 28]. Available from: https://www.opennotes.org/history/.

[4] Peck P Torous J Shanahan M Fossa A Greenberg W Patient access to electronic psychiatric records: a pilot study. Heal Policy Technol [Internet]. (2017) 6(3):309–15. 10.1016/j.hlpt.2017.06.003.

[5] Petersson L Erlingsdóttir G Open notes in swedish psychiatric care (part 2): survey among psychiatric care professionals. J Med Internet Res (2018) 20(6):e10521. 10.2196/10521 PMC6035347 29929946

[6] Open Notes Open Notes Mental Health [Internet]. (2017). [cited 2017 Nov 8]. Available from: https://www.opennotes.org/tools-resources/for-health-care-providers/mental-health.

[7] Denneson LM Pisciotta M Hooker ER Trevino A Dobscha SK Impacts of a web-based educational program for veterans who read their mental health notes online. J Am Med Inf Assoc (2018) 0: (0):1–6. 10.1093/jamia/ocy134 PMC7647153 30445648

[8] Petersson L Erlingsdóttir G Open Notes in Swedish Psychiatric Care (Part 1): survey among psychiatric care professionals. JMIR Ment. Heal [Internet]. (2018) 5(1):e11. 10.2196/mental.9140 PMC5816262 29396386

[9] Strudwick G Clark C Sanches M Strauss J Predictors of mental health professionals’ perceptions of patient portals. AMIA Annu Symp Proc (2018) 2018:989–97. PMC6371312 30815142

[10] Dobscha SK Denneson LM Jacobson LE Williams HB Cromer R Woods S VA mental health clinician experiences and attitudes toward OpenNotes. Gen Hosp. Psychiatry [Internet]. (2016) 38:89–3. 10.1016/j.genhosppsych.2015.08.001 26380876

[11] Kipping S Stuckey MI Hernandez A Nguyen T Riahi S A web-based patient portal for mental health care: benefits evaluation. J Med Internet Res (2016) 18(11):1–9. 10.2196/jmir.6483 PMC5131190 27852556

[12] Open Notes Mental Health Toolkit [Internet]. (2017). [cited 2017 Sep 20]. Available from: https://www.opennotes.org/tools-resources/for-health-care-providers/mental-health/

